# Clinicopathological characteristics of oral squamous cell carcinoma in Northern Norway: a retrospective study

**DOI:** 10.1186/1472-6831-14-103

**Published:** 2014-08-18

**Authors:** Oddveig G Rikardsen, Inger-Heidi Bjerkli, Lars Uhlin-Hansen, Elin Hadler-Olsen, Sonja E Steigen

**Affiliations:** 1 Department of Otorhinolaryngology, University Hospital of North Norway, Tromsø, Norway; 2 Department of Medical Biology - Tumour Biology Research Group, UiT The Arctic University of Norway, Tromsø, Norway; 3 Diagnostic Clinic – Clinical Pathology, University Hospital of North Norway, Tromsø, Norway

**Keywords:** Oral squamous cell carcinoma, Smoking, Alcohol, Primary site, Gender

## Abstract

**Background:**

The main aim of the study was to evaluate if patients with oral squamous carcinomas in Northern Norway differ from patients in other countries with regard to clinicopathological characteristics and also study the influence of risk factors. Such a comparison is of demographical interest, and also important for the interpretation of result from studies on prognostic biomarkers.

**Methods:**

We describe clinicopathological characteristics of 133 North Norwegian patients diagnosed with squamous cell carcinoma of the oral cavity in the period 1986–2002, and evaluate the significance of different risk factors.

**Results:**

The cohort consisted of 69 men and 64 women, giving male/female ratio of 1.1. Forty-seven of the 133 patients (35%) died of the disease within 5 years from diagnosis. There was no significant difference between the genders concerning time to disease specific death, even though men both smoked and drank more alcohol than women. As expected, the strongest predictors for disease specific death were tumour size and the presence of regional lymph node metastasis. We also found that heavy smokers and drinkers presented with more advanced disease, more often localized to the floor of mouth compared to non-smoking and abstinent patients, who more often presented with tumours of the mobile tongue*.*

**Conclusions:**

Our results correlate well with previously published clinicopathological data on comparable cohorts, which is important when considering the applicability of results from biomarker studies performed on this material compared to other cohorts, and vice versa.

## Background

Squamous cell carcinoma (SCC) accounts for more than 90% of the malignant neoplasm’s in the oral cavity, which includes tongue, floor of mouth, buccal mucosa, alveolar rim and the hard and soft palate [1,2]. Globally, about 275,000 new cases of oral SCC (OSCC) are diagnosed each year [3], but the incidence of OSCC show large geographical variations*.* In the southern part of Asia and in Latin America the incidence of OSCC is about 20 fold higher than in Northern Europe, and is actually the most common cancer among the male population in some of the high-risk areas in Asia [3].

The lifetime risk of developing oral or oropharyngeal cancer in Europe is estimated to be 1.85% for men and 0.37% for women [3]. Age-standardized incidence (rates per 100 000) for lip and oral cancer in Northern Europe is 5.1 for men and 2.5 for women [4]. In Norway the equivalent numbers are 4.3 and 2.5. In these numbers SCC of the lip accounts for approximately 40% of the cases [5]. The incidence rates are higher in Western and Eastern Europe than in northern or southern European countries; France and Hungary presenting the highest numbers, Greece and Cyprus the lowest [6,7]. In Northern France, the oral cavity and oropharynx constitute the second most common cancer sites in men, after lung [8]. The large geographical variations in incidence are mainly explained by cultural differences which is influenced by the exposure to risk factors [2]. The two best known risk factors in the Western countries are tobacco and alcohol abuse, which act strongly synergistically, and are estimated to account for up to 75% of the disease burden in this part of the world [7,9-11]. Koch et al. reported that cigarette smoking increased the risk for OSCC 1.9 times in men and 3 times in women. For persons who stop smoking, the risk falls to non-smoker level in 10 years. Daily drinkers (2 units/day) are reported to have an increased oral cancer risk of 1.7, heavy drinkers up to 3. For persons who smoke *and* drink alcohol daily, the odds ratio are reported to be as high as 35 [12,13]. In Southern Asia the chewing of betel quid and areca nuts explains the high incidence [14,15]. The exposure to various risk factors also has impact on the primary site of OSCC, betel quid and areca nut chewing mainly disposing for tumours arising in the buccal mucosa, while cigarette smoking and alcohol are disposing for tumours in the floor of mouth [1,12,16-18].

The most widely used classification-system for describing the anatomical extent of the disease is the Union for International Cancer Controls TNM-system [19] which grades primary tumour size and invasion features (T), regional lymph node spread (N) and the presence of distant metastasis (M). Survival time of the OSCC patients is strongly associated with the TNM-stage, and the TNM-classification system is still the most important guide for treatment stratification in clinical practice [20,21]. By morphological assessment, tumours are classified based on the cancer cells differentiation into well, moderately and poorly differentiated carcinomas [1]. Some report that patients with well differentiated tumours live longer than patients with low differentiated tumours [22,23], whereas other report poor correlation between outcome and tumour grade [24,25].

The acquired knowledge about the impact of human papilloma virus (HPV) infection in oropharyngeal tumours highlights the importance of distinguishing oropharyngeal tumours from tumours in the oral cavity. An increasing number of the tumours arising in the oropharynx are thought to be HPV-driven. The behaviour and treatment response of these tumours differ to such a degree from HPV negative tumours that an increasing number of scientists claim that they should be considered as two different enteties [20,26]. In the oral cavity, though, HPV-related tumours are uncommon, and the HPV-status of the tumour has little impact on outcome [27-29]. A tumour cell infected with an oncogenic HPV-type, overexpress the tumour suppressor protein p16^INK4a^ due to retinoblastoma (Rb) gene inactivation by the E7 oncoprotein [30]. In clinical practice the HPV status of a tumour is often determined indirectly by immunohistochemical identification of overexpression of the p16 protein [31,32].

In the present study we present a cohort of 133 OSCC patients from Northern Norway. The population of Northern Norway may be exposed to other risk factors than people in other cohorts. The main aim of the study was therefore to evaluate if patients with OSCC in Northern Norway differ from patients in other countries with regard to clinicopathological characteristics. Such a comparison is not only of demographical interest, but is also important for the interpretation of result from studies on prognostic biomarkers performed on this cohort to other populations, and for implementation of results from studies on other populations around the world to our patients.

## Methods

### Specimens

The cohort described in this paper consists of 133 patients collected from the archives of the Department of Clinical Pathology, University Hospital of North Norway. They represent all patients diagnosed at the department with primary SCC of the oral cavity (ICD-O, C02-C06) [33] in the period 1986–2002. Second primaries were excluded.

The relevant clinical data and the tumours’ TNM-classification [19] were retrieved from patients files, including pathology reports and Statistics of Norway, Cause of Death Registry. The N-stage was based on clinical and radiological findings. The study was approved by the Regional committees for Medical and Health Research Ethics, Northern Norway (No. 22/2007).

### Immunhistochemical staining of p16

Tissue microarray (TMA) blocks containing 2 cores of 0.6 mm from representative tumour tissue from all patients were sectioned at 4 μm and transferred to Superfrost + slides. The immunohistochemical staining for p16 was done in the automated slide stainer Ventana Benchmark, XT (Ventana Medical Systems, Inc., Roche, Tucson Arizona, USA) at the Department of Clinical Pathology, University Hospital of North Norway, using the same protocols, and positive and negative controls as in the clinical routine. The antibody used was a prediluted mouse anti-p16, clone E6H4, Ventana, recognizing the tumour suppressor p16^INK4a^ protein. The slides were pre-treated with cell conditioning 1 (CC1) at 95°C for 36 minutes, and the antibody incubation time was 28 minutes.

The staining was recorded as percentage of tumour cells stained; (1 (<10%), 2 (10-50%), 3 (51-80%) and 4 (>80%), and was performed by one experienced pathologist (SES) and one head and neck surgeon (OR). The scoring was easy to perform, as the majority of the tissue samples were either completely negative, or clearly positive. In cases of disagreement the score was discussed and an consensus was achieved.

### Statistics and analyses

The statistical analyses were performed using IBM SPSS statistics for Windows (IBM Corporation Armonk, New York), version 21. Associations between different categorical variables were assessed with Pearson’s chi-square test and Pearson correlation. Comparing of means was done using the ONE-Way ANOVA-test. Univariate analyses of time from diagnosis to death were performed using the Kaplan-Meier method, and differences between categories were estimated by the log-rank test, with the date of diagnosis as starting point. The influence of covariates on patient survival was analysed by multivariate analysis (proportional hazard method). The last day of follow-up was 01.01.2012. All results were considered significant if p ≤ 0.05.

To control for variation due to age the patients were divided into five groups (≤50 years, 51–60 years, 61–70 years, 71–80 years and ≥81 years). We also constructed a variable called “Integrading risk factors” (IRF) that combine the factors smoking and alcohol, and a factor called “Stage of disease” (SOD) that combined the T, N and M-stage based on the American Joint Comitee on Cancer (AJCC) staging system [34].

## Results

### Gender and age

The cohort consisted of 52% men and 48% women, giving a male/female ratio of 1.1. All clinicopathological variables defined by gender and survival analyses are summarized in Table 1. The mean age at diagnosis was 66.3 years, 64.9 for men (range 29–93) and 67.7 (range 27–90) for women. The mean overall survival from diagnosis was 56.7 months for men and 88.3 months for women, a difference that was statistically significant (p = 0.021).

**Table 1:** Clinicopathiological variables defined by gender and disease specific death (DSD)

			**Difference**		**DSD**	
	**Men**	**Women**	**Gender**	**All patients**	**N**	
	**(n = 69)**	**(n = 64)**	p-value*	**(n = 133)**	**(% of total)**	DSD p*
**Age**						
≤50 years	8 (12%)	9 (14%)	p = 0.405	17 (13%)	4 (24%)	0.769
51-60 years	19 (28%)	11 (17%)		30 (23%)	12 (40%)	
61-70 years	18 (26%)	14 (22%)		32 (24%)	14 (44%)	
71-80 years	15 (22%)	15 (23%)		30 (23%)	9 (30%)	
≥81 years	9 (13%)	15 (23%)		24 (18%)	8 (33%)	
**Tumor differentiation**						
Verrocous	1 (1%)	4 (6%)	p = 0.474	5 (4%)	0	0.142
Well	28 (41%)	28 (44%)		56 (42%)	17 (30%)	
Moderate	34 (49%)	27 (42%)		61 (46%)	25 (41%)	
Poor	6 (9%)	5 (8%)		11 (8%)	5 (45%)	
**Tumor stage**						
T1	19 (28%)	24 (39%)	p = 0.335	43 (32%)	8 (19%)	**<0.001**
T2	25 (36%)	21 (33%)		46 (35%)	11 (24%)	
T3	4 (6%)	7 (11%)		11 (8%)	7 (64%)	
T4	18 (26%)	9 (14%)		27 (20%)	17 (63%)	
Unknown	3 (4%)	3 (5%)		6 (5%)	4 (67%)	
**Lymph node status**						
N0	42 (61%)	40 (63%)	p = 0.200	82 (62%)	21 (26%)	**<0.001**
N +^a^	21 (30%)	13 (20%)		34 (26%)	21 (62%)	
Unknown	6 (9%)	11 (17%)		17 (13%)	5 (29%)	
**SOD**^**b**^						
Stage I	15 (22%)	17 (27%)	p = 0.316	32 (24%)	4 (13%)	**<0.001**
Stage II	19 (28%)	16 (25%)		35 (26%)	7 (20%)	
Stage III	9 (13%)	11 (17%)		20 (15%)	10 (50%)	
Stage IV	20 (29%)	10 (16%)		30 (23%)	21 (70%	
Unknown	6 (9%)	10 (16%)		16 (12%)	5 (31%)	
**Primary site**						
Mobile tounge	34 (49%)	27 (42%)	**p = 0.038**	61 (46%)	18 (30%)	0.548
Floor of mouth	21 (30%)	9 (14%)		30 (22%)	12 (40%)	
Alveolar rim	9 (13%)	15 (23%)		24 (18%)	8 (33%)	
Buccal mucosa^c^	4 (6%)	7 (11%)		18 (14%)	9 (50%)	
Palate^c^	1 (1%)	3 (5%)				
Other^c^	0	3 (5%)				
**Smoking**						
Never	8 (12%)	27 (42%)	**p < 0.001**	35 (26%)	9 (26%)	0.566
Previous smoker	14 (20%)	6 (9%)		20 (15%)	7 (47%)	
Smoker	41 (59%)	18 (28%)		59 (44%)	24 (41%)	
Unknown	6 (9%)	13 (20%)		19 (14%)	7 (37%)	
**Alcohol consumption**						
Never	5 (7%)	23 (36%)	**p < 0.001**	28 (21%)	6 (21%)	**0.002**
< once a week	27 (39%)	20 (31%)		47 (35%)	16 (34%)	
> once a week	9 (13%)	1 (2%)		10 (8%)	2 (20%)	
Daily	8 (12%)	3 (5%)		11 (8%)	7 (64%)	
Unknown	20 (29%)	17 (27%)		37 (28%)	16 (43%)	
**IRF**^d^						
Non-smoker/non-drinker	10 (14%)	30 (47%)	**p < 0.001**	40 (30%)	10 (25%)	0.120
Smoker/light drinker^e^	22 (32%)	13 (20%)		35 (26%)	12 (34%)	
Smoker/heavy drinker^e^	17 (25%)	4 (6%)		21 (16%)	9 (43%)	
Unknown	20 (29%)	17 (27%)		37 (28%)	16 (43%)	
**P16-staus**^f^						
Positiv	6 (10%)	4 (7%)	p = 0.625	100 (88%)	38 (33%)	0.791
Negative	54 (90%)	50 (93%)		10 (9%)	4 (4%)	

^a^includes N1, N2a, N2b, N2c and N3.

^b^SOD = Stage of Disease.

^**c**^In the further analyses, these three are merged.

^d^IRF = Intergrading risk factors.

^e^light drinker = less than once a week; heavy drinker = more than once a week or daily.

^f^N = 114 in the p16 analysis.

*p < 0.05 was regarded as statistically significant, and highlighted in boldface when present.

Forty-seven (35%) of the 133 patients (35%) died of the disease within 5 years from diagnosis, 41% of the men and 30% of the women. Although the women in general lived longer than the men, the difference in 5 years disease specific survival was not statistically significant (p = 0.189). There was a tendency that women were diagnosed with smaller tumours (lower T-stage) than men (p = 0.078).

Nine patients (7%) were younger than 45 years at the time of diagnosis, which completely coincide with what has been reported globally [6]. Three of these young patients died of the disease.

**Figure 1:**
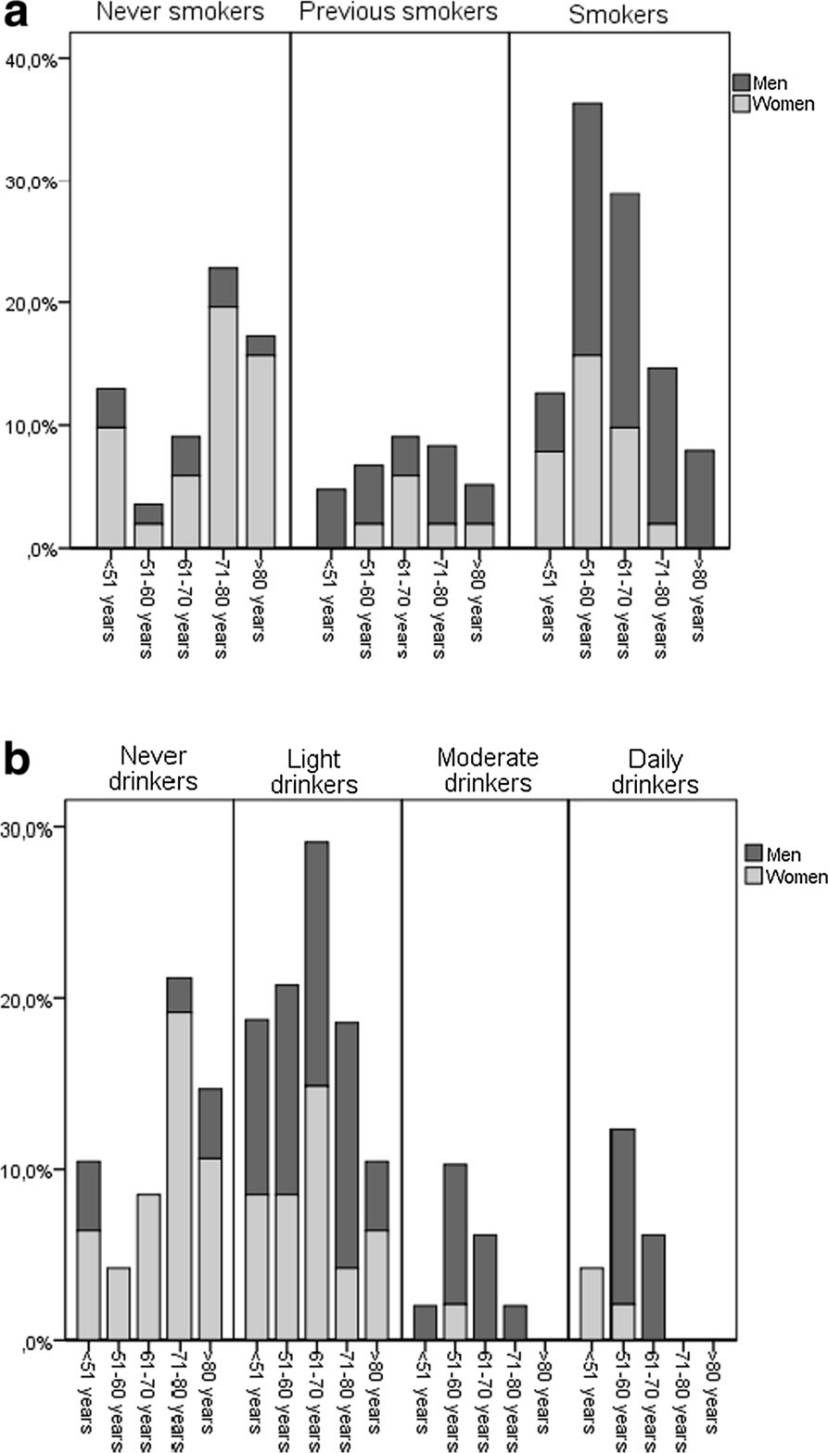
**A graphical illustration of the relation between smoking (a) and alcohol consumption (b) in the different age-groups.** The columns are split by gender.

The risk of dying of the disease within 5 years was the same in all age-groups, see Table 1. There was no significant association between age and primary site, grade of tumour differentiation, p16-status, tumour size (T) *nor* the presence of regional metastasis (N). However, the youngest and eldest patients smoked less tobacco (p = 0.008), and consumed significantly less alcohol (p = 0.002) compared to the middle-aged, see Figure 1a and b.

### Anatomic site and classification

The majority of the tumours arose in the mobile tongue (46%), floor of mouth (23%) and alveolar ridge (18%) (Table 1). There was a significant correlation between gender and primary site (p = 0.015), men accounting for 70% of the tumours in the floor of mouth, while tumours of the alveolar rim being most frequently diagnosed in women (63%) (Table 2).

**Table 2:** Smoking and alcohol consume in relation to primary site

**Men**	**Mobile tounge**	**Floor of mouth**	**Alveolar rim**	**Other**^**a**^	p*
Total	34 (49%)	21 (30%)	9 (13%)	5 (7%)	
**Smoking**					
Never	7 (21%)	1 (5%)	0	0	**p = 0.047**
Previous	8 (24%)	2 (10%)	2 (22%)	2 (40%)	
Smoker	17 (50%)	17 (81%)	6 (67%)	1 (20%)	
Unknown	2 (6%)	1 (5%)	1 (11%)	2 (40%)	
**Drinking**					
Never	4 (12%)	1 (5%)	0	0	p = 0.061
< once a week	18 (53%)	4 (19%)	4 (44%)	1 (20%)	
> once a week	2 (6%)	6 (29%)	1 (11%)	0	
Daily	2 (6%)	5 (24%)	0	1 (20%)	
Unknown	8 (24%)	5 (24%)	4 (44%)	3 (60%)	
**Women**	**Mobile tounge**	**Floor of mouth**	**Alveolar rim**	**Other**^**a**^	p*
Total	27 (42%)	9 (14%)	15 (23%)	13 (20%)	
**Smoking**					
Never	17 (63%)	1 (11%)	6 (40%)	3 (23%)	**p = 0.007**
Previous	3 (11%)	1 (11%)	1 (7%)	1 (8%)	
Smoker	5 (19%)	5 (56%)	1 (7%)	7 (54%)	
Unknown	2 (7%)	2 (22%)	7 (47%)	2 (15%)	
**Drinking**					
Never	14 (52%)	0	6 (40%)	3 (23%)	**p = 0.017**
< once a week	9 (33%)	5 (56%)	2 (13%)	4 (31%)	
> once a week	0	1 (11%)	0	0	
Daily	1 (4%)	0	2 (13%)	2 (15%)	
Unknown	3 (11%)	3 (33%)	4 (27%)	4 (31%)	

*p < 0.05 was regarded as statistically significant, and highlighted in boldface when present.

^a^includes buccal mucosa, palate and unspecified site in the oral cavity.

Most of the tumours were well (41%) or moderately differentiated (46%) and 4% of the tumours showed a verrucous growth pattern. There was a higher occurrence of lymph node metastasis among the poorly differentiated tumours, but this was not statistical significant.

### Smoking and alcohol

Smoking and drinking habits of the 133 patients are listed in Table 1. As this was a retrospective study, it was not possible to specify the amount of tobacco and alcohol use in pack-years or units due to unstandardized reporting in the patients’ files. As expected, there was a strong correlation between smoking and drinking (p = 0.01), and a significant predominance of men among both smokers and drinkers (p < 0.001). Compared to non-smokers and abstinent patients, smokers and drinkers presented with significantly larger tumours (p = 0.002 for smoking, p = 0.017 for drinking) and a higher frequency of regional node spread of the disease (p < 0.001 for smoking, p = 0.002 for drinking). There was a highly significant correlation between smoking and drinking and SOD (p < 0.001).

For the whole cohort, there was also a highly significant correlation between both smoking and drinking and primary site (p < 0.001 for both). 36% of the patients with tumours in the mobile tongue were smokers compared to 73% of the patients with tumours arising in floor of mouth. Only 8% of the patients with tongue cancers were recorded to be drinking more than once a week or daily compared to 40% of patients with cancers in the floor of the mouth. When stratified on gender, the correlation between smoking and primary site was statistically significant for both men and women (p = 0.047 and p = 0.007 respectively), but only women showed a statistically significant correlation between drinking habits and primary site (p = 0.017 vs. 0.061 for men) (Table 2).

### HPV-related tumours

p16 expression is often used as an indirect indicator of HPV infection in the tumour cells in clinical practice [32]. Of the 114 patients that we assessed for p16 expression in their primary tumour, 9% were p16 positive (Table 1). For 16 of the patients, tissue from lymph node metastasis were also stained, which showed that p16 expression in the primary tumours and the metastatic foci correlated completely (2 positive/14 negative). There was no correlation between p16-status and 5 years survival rate, site of tumor or any of the other clinicopathological variables.

### Mortality

**Figure 2:**
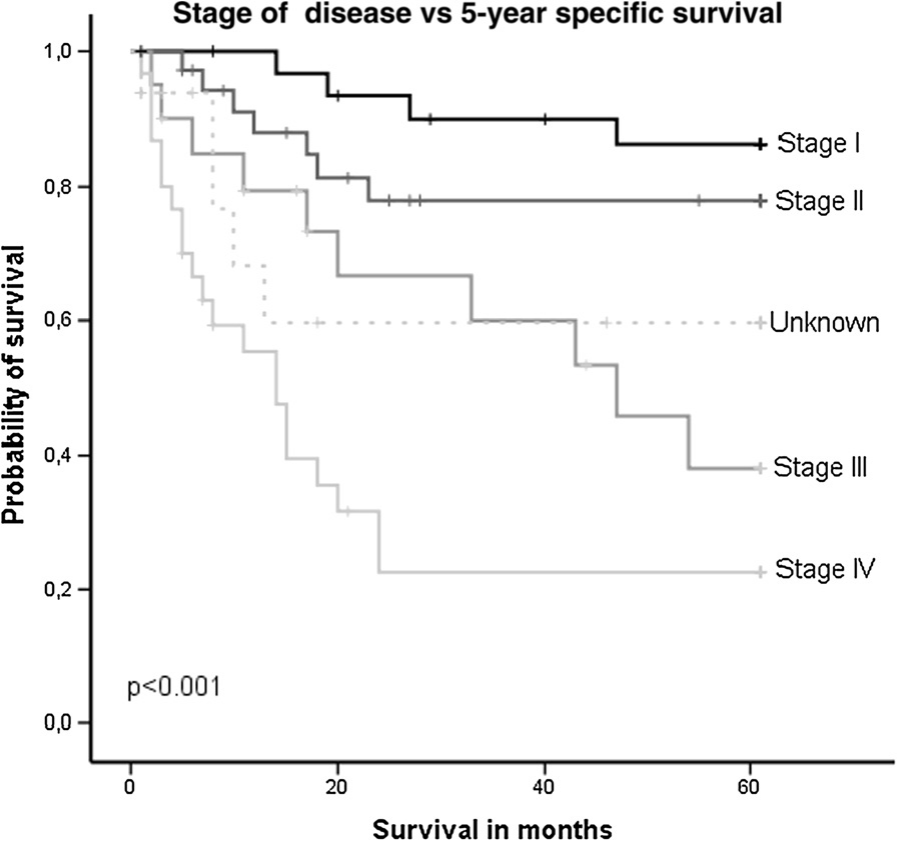
Kaplan Meier curve illustrating the disease specific survival in relation to stage of disease (SOD).

As expected, we found that tumour size, presence of regional lymph node metastasis, as well as the compounded factor SOD, correlated with DSD (p < 0.001, p = 0.001 and p < 0.001, respectively, Figure 2). Furthermore, high alcohol consumption increased the 5-years risk of dying of the disease, both for the whole cohort (p = 0.002) and when stratified on men and women (p = 0.039 and p = 0.001). None of the other variables showed any statistically significant association with 5-year DSD.

In a multivariate Cox regression analyses, including primary site, SOD, smoking and drinking, only SOD was significant for the whole cohort. Though, when evaluating genders separately, also drinking habits and the Integrading risk factors (IRF) variable were independent prognostic factors in women (p = 0.01 and p = 0.02, respectively).

## Discussion

We have demonstrated that our North Norwegian cohort of patients with oral cancer is comparable with other groups investigated in international studies in most aspects.

As reported in other studies, the majority of the patients presented with well or moderately differentiated tumours, most frequently located in the mobile tongue, floor of mouth and alveolar rim [1,35]. As reported in a North American study by Koch et al., we found that the smokers and drinkers were diagnosed between the fifth and seventh decades of life [12]. There were less smokers and drinkers among the youngest and oldest individuals, who frequently presented tumours in the mobile tongue. This is also consistent with previous published data [12]. Tumours arising in the floor of mouth were by far more frequent in male patients with a high consume of both tobacco and alcohol (Table 2). This has also been reported by others, and has been explained by the accumulation of carcinogens in the mucus in the floor of the mouth [1,12].

Our results indicate that alcohol is a more potent risk factor for development of OSCC than smoking. La Vecchia et al. [7] claims that alcohol has a major relevance in defining the individual risk of oral cancer. They describe a rising trend in oral cancer up to the mid 1990 in Europe, reflecting the expanding epidemic of all tobacco-related neoplasms in those countries. Though, over the last decades there has been a fall in lung cancer, but a persistent unfavourable trend in oral cancer in males from the UK, essentially attributable to changes in alcohol habits. Furthermore, we found that the correlation between drinking and DSD was stronger in women than in men. In the multivariate analysis, increased alcohol consumption was a significant risk factor for disease specific death in women, but not in men or the whole cohort. Other studies have also claimed that women are more vulnerable to the harmful effects of alcohol than men [36].

In our study we found a male/female ratio of 1.1, which is somewhat lower than reports from other countries in Northern Europe, like Denmark and the UK, where the ratio vary between 1.3 to 2.0 [4,6,37]. There is however a global trend of decreasing differences between genders [3,8]. The Cancer registry of Norway, which publish data in cancer incidence, mortality, survival and prevalence in Norway, reports (for 1995) a 2.1:1 ratio for men versus women for cancer categorized as mobile tongue and mouth (3.0 and 1.4/100 000) (ICD-O, C02-C06) [33]. However, for the three northernmost counties in Norway, representing our impact area, the ratio in the national statistics was 1.3:1 (2.6 and 2.0/100 000) which is in accordance with the distribution found in our study. Thus the North Norwegian population does indeed seem to have a slightly lower ratio between men and women than reported elsewhere.

According to available data for alcohol consumption in Norway in 1999, men consumed 4.82 litres of pure alcohol per year (35% classified as hard liquor), and women 1.94 (20% hard liquor) [38]. The numbers in the three northernmost counties did not differ much from the rest of Norway [39]. There are no reliable studies reporting amount and type of alcohol consumed in men and women in the different regions of Norway, although many speculate that moonshine (illegally distillate spirits) is consumed to a somewhat larger degree by both genders in the northern part of Norway. Several studies have shown that hard liquor have a larger impact on OSCC-development than wine and beer [13,40,41].

In our material, which is not adjusted for age, there was a tendency for an increased proportion of women in the higher age-groups. Statistics of Norway reveal that in 1995 women in Norway had an overall 6 year longer life expectancy then men [42]. This longer life-expectancy for women, and the lack of adjustment for this in our material, might explain that the male/female ratio was somewhat low, despite the fact that men drank significantly more than women.

The 5-year risk for DSD was 35% for the whole cohort, 41% for the men and 30% for the women respectively, which is somewhat lower than often reported [43,44]. The difference between men and women was not statistically significant. The majority of the patients in our study were diagnosed with an early stage disease, presenting with small (T1 (30%) or T2 (33%)) primary tumours and 82 out of 133 patients (62%) without detectable lymph node metastasis. This may in part be due to a well-functioning health-care system providing easy access and good treatment prospects. Women were in general diagnosed with smaller tumours than men, which suggest that they are diagnosed at an earlier stage of tumour development. The longer survival could partly be a result of earlier diagnosis, and not the fact that women survive longer than men (lead time bias).

In 2001, Mork et al. [28], reported the proportion of HPV-related tumours in the oral cavity of Scandinavian head and neck-cancer patients to be 6.8%. This correlates well with our numbers where 8.7% of the tumours were p16 positive. However, in the study by Mork, all of the HPV-positive tumours arose in the mobile tongue, giving a frequency of 14% at this specific site. In our material there was a small, but not statistically significant difference in p16-positivity in tumours of the mobile tongue (11%) compared to the rest of the oral cavity (7%). In most studies, HPV-positivity has been found to correlate with a favourable prognosis for carcinomas in the oropharynx, but in line with our results, such a correlation are seldom reported for carcinomas in the oral cavity or other sub sites in the head and neck region [29,45]. There has also been reported a higher degree of HPV-negative carcinomas among smokers [12], but there was no such tendency in our material.

## Conclusion

Our study confirms that the North Norwegian OSCC patient is representative of the general OSCC patient in Europe, both regarding primary site, the stage of the disease and associations with risk factors such as smoking and drinking. This is important when considering the applicability of results from biomarker studies performed on this material to other cohorts. Likewise, results from other studies can be applicable for the population of Northern Norway.

## Competing interests

The authors declare that they have no competing interests.

## Authors’ contributions

OR has contributed to the conception and design of the study, collected data, analysed and interpreted the data, drafted and later revised the manuscript. IHB has participated in the design of the study, interpreted the data, and revised the manuscript. LUH has been central in the conception and design of the study, interpreting the data, and revised the manuscript. EHO has participated in the design of the study, critically interpreted the data, and revised the manuscript. SES has contributed to the conception and design of the study, analysed and interpreted the data, drafted and revised the manuscript. All authors have given their final approval of the version to be published.

## Pre-publication history

The pre-publication history for this paper can be accessed here:

http://www.biomedcentral.com/1472-6831/14/103/prepub
